# Affinity of ^nat/68^Ga-Labelled Curcumin and Curcuminoid Complexes for β-Amyloid Plaques: Towards the Development of New Metal-Curcumin Based Radiotracers

**DOI:** 10.3390/ijms17091480

**Published:** 2016-09-06

**Authors:** Sara Rubagotti, Stefania Croci, Erika Ferrari, Michele Iori, Pier C. Capponi, Luca Lorenzini, Laura Calzà, Annibale Versari, Mattia Asti

**Affiliations:** 1 Nuclear Medicine Unit, Oncology and Advanced Technologies Department, Arcispedale Santa Maria Nuova-IRCCS, 42123 Reggio Emilia, Italy; rubagotti.sara@asmn.re.it (S.R.); iori.michele@asmn.re.it (M.I.); capponi.piercesare@asmn.re.it (P.C.C.); versari.annibale@asmn.re.it (A.V.); asti.mattia@asmn.re.it (M.A.); 2 Clinical Immunology, Allergy, and Advanced Biotechnologies Unit, Diagnostic Imaging and Laboratory Medicine Department, IRCCS-Arcispedale Santa Maria Nuova, 42123 Reggio Emilia, Italy; croci.stefania@asmn.re.it; 3 Department of Chemical and Geological Sciences, University of Modena, 41125 Modena, Italy; 4 Health Sciences and Technologies-Interdepartmental Center for Industrial Research (HST-ICIR), University of Bologna, 40126 Ozzano Emilia, Italy; luca.lorenzini8@unibo.it (L.L.); laura.calza@unibo.it (L.C.)

**Keywords:** curcumin, alzheimer disease, Gallium-68, β-amyloid, curcuminoid complexes, fluorescence

## Abstract

Curcumin derivatives labelled with fluorine-18 or technetium-99m have recently shown their potential as diagnostic tools for Alzheimer’s disease. Nevertheless, no study by exploiting the labelling with gallium-68 has been performed so far, in spite of its suitable properties (positron emitter, generator produced radionuclide). Herein, an evaluation of the affinity for synthetic β-amyloid fibrils and for amyloid plaques of three ^nat/68^Ga-labelled curcumin analogues, namely curcumin curcumin (CUR), bis-dehydroxy-curcumin (bDHC) and diacetyl-curcumin (DAC), was performed. Affinity and specificity were tested in vitro on amyloid synthetic fibrils by using gallium-68 labelled compounds. Post-mortem brain cryosections from Tg2576 mice were used for the ex vivo visualization of amyloid plaques. The affinity of ^68^Ga(CUR)_2_^+^, ^68^Ga(DAC)_2_^+^, and ^68^Ga(bDHC)_2_^+^ for synthetic β-amyloid fibrils was moderate and their uptake could be observed in vitro. On the other hand, amyloid plaques could not be visualized on brain sections of Tg2576 mice after injection, probably due to the low stability of the complexes in vivo and of a hampered passage through the blood–brain barrier. Like curcumin, all ^nat/68^Ga-curcuminoid complexes maintain a high affinity for β-amyloid plaques. However, structural modifications are still needed to improve their applicability as radiotracers in vivo.

## 1. Introduction

The accumulation of amyloid-β (Aβ) aggregates as soluble oligomers and senile plaques in the brain are key landmarks for Alzheimer’s disease (AD) and their presence can be exploited as a selective target for diagnostic and therapeutic drugs [1,2]. Recently, several studies revealed that (1*E*,6*E*)-1,7-Bis(4-hydroxy-3-methoxyphenyl)-1,6-heptadiene-3,5-dione also known as curcumin showed high affinity for Aβ-amyloid plaques in vitro and in vivo and anti-AD properties due to its ability to bind and subsequently disrupt the aggregation of amyloid peptide and already formed fibrils as well [3,4]. Due to this finding, radio-labelled curcumin derivatives could be potential biomarkers for imaging of Alzheimer’s disease by means of nuclear medicine imaging techniques. The qualitative and quantitative detection of amyloid deposits could likely play an important role for the early diagnosis and for the monitoring of the progression of Alzheimer’s disease as Aβ-fibrils’ accumulation occurs early before clinical symptoms appear. Curcumin is a phyto-compound extracted from the roots of *Curcuma longa* L. with strong antioxidant and anti-inflammatory properties that exhibits a pH and solvent dependent keto-enol tautomerism. Moreover, curcumin is a fluorochrome emitting in the visible spectrum between 450 and 650 nm. Unfortunately, native curcumin exhibits poor physiological properties such as low bioavailability, poor water solubility and low stability [5]. Hence, structural modifications are needed both for stabilizing the molecule as well as for labelling the stabilized derivatives with the proper radionuclide. A first way for achieving these aims was obtained by simple addition of a pendant arm with a suitable leaving group to the curcumin structure with the main purpose of allowing the labelling with fluorine-18 [6,7]. Recent studies provided for more complicated modifications of the backbones in order to obtain higher stability of the precursor as well as an easier way for introducing the fluorine-18 atom [8]. In the last years, synthesis, radio-labelling and pre-clinical applications of this class of compounds were investigated generally achieving positive results in vitro but failing in their performances in vivo. 

A second way was explored by studying the properties of the curcumin/curcuminoids complexes as these compounds often exhibit higher solubility in aqueous media than free ligands and the coordinating metal could be quite easily selected in the plethora of the radiometals suitable for nuclear medicine applications. In fact, by using the curcuminoids as complexing agents, it is possible both to improve the physiological properties of the derivative and to introduce a radionuclide useful for imaging purposes. In a recent publication, curcumin was employed as OO bidentate ligand in some complexes with a technetium-99m tricarbonyl core. This class of radiotracers showed a high affinity for Aβ-amyloid plaques ex vivo on a section of brain tissue of a neuropathologically diagnosed AD patient [9]. 

**Figure 1:**
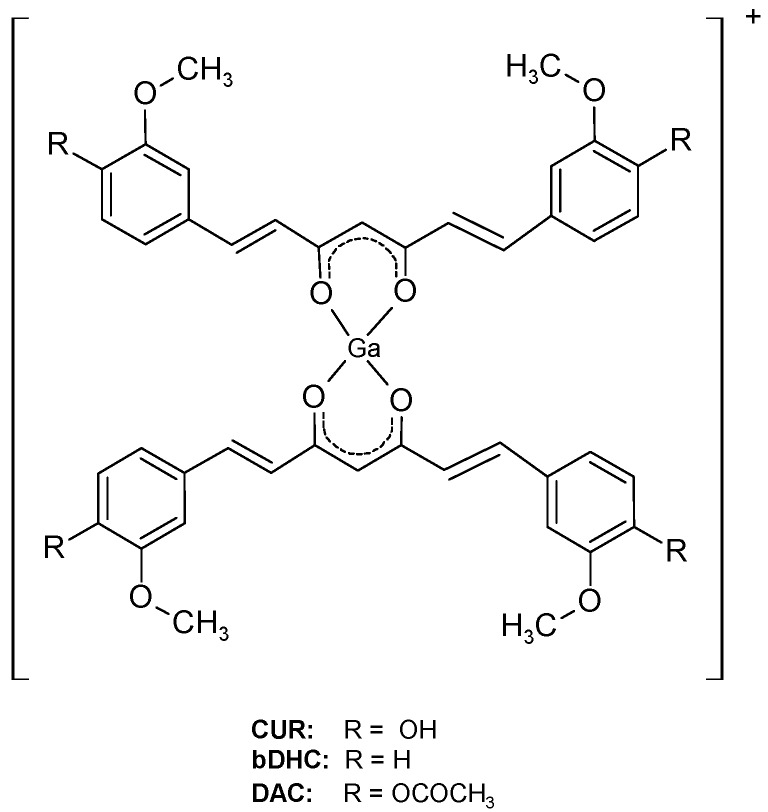
Chemical structure of investigated Ga–curcuminoids complexes. The apical positions of the pseudo-octahedral coordination of the metal core are likely occupied in solution by labile ligands such as Cl^−^ anions or water molecules.

If compared with fluorine-18 and technectium-99m, gallium-68 exhibits advantageous features being a generator produced positron emitter radionuclide with physical and chemical characteristics suitable for diagnostic nuclear medicine and direct labelling of biomolecules (89% β^+^, maximum energy = 1.92 MeV; T_1/2_ = 67.7 min). Speculating on the fact that curcumin complexes appear to maintain the properties of free curcumin regarding the affinity to amyloid plaques, three new gallium-68 labelled curcuminoids complexes, namely ^68^Ga(CUR)_2_^+^, ^68^Ga(DAC)_2_^+^, ^68^Ga(bDHC)_2_^+^ whose general structure is reported in Figure 1, were recently synthesized and characterized [10].

The aim of the following study is to investigate the biological properties in vitro and in vivo of the three ^nat/68^Ga-curcuminoids complexes exploiting both the intrinsic fluorescence of these derivatives and the radioactive properties of gallium-68. The results will give insight into the possibility to employ these compounds as radiotracers for monitoring the presence of Aβ-amyloid plaques in vivo by positron emission tomography (PET).

## 2. Results

### 2.1. Radiosynthesis

**Figure 2:**
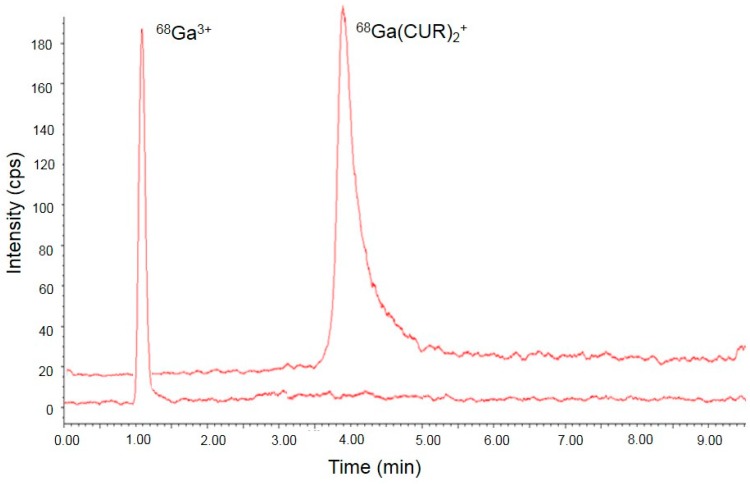
Comparison between radio-HPLC (high-performance liquid chromatography) chromatograms obtained from ^68^Ga(CUR)_2_^+^ complex and free-^68^Ga^3+^ attesting the completion of the reaction and the radiochemical purity of the product.

Synthesis of ^68^Ga-labelled curcuminoids was accomplished in 10 min in quantitative yield and radiochemical purity >95%. Batches of ca. 100 MBq with a specific activity of ca. 2 MBq/nmol were synthesized for the in vitro studies. When necessary, the batches were diluted in a Dimethyl sulfoxide(DMSO)/H_2_O 10/90 solution. A paradigmatic high-performance liquid chromatography (HPLC) chromatogram of ^68^Ga(CUR)_2_^+^ and free-Ga^3+^ was shown in Figure 2.

### 2.2. In Vitro Study

**Figure 3:**
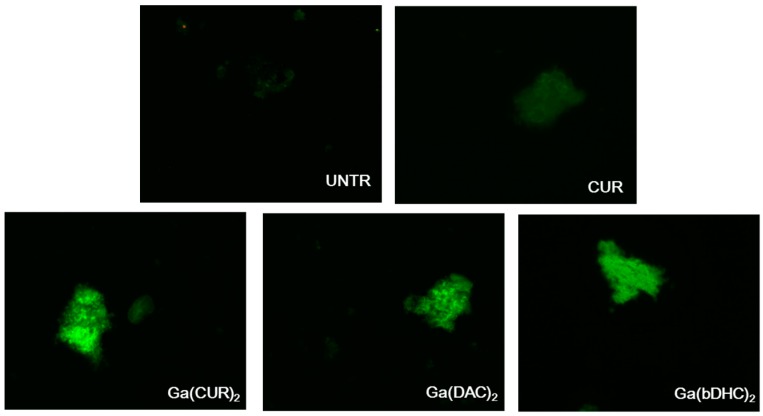
Images of the fluorescence associated to synthetic untreated Aβ-amyloid plaques (UNTR) and synthetic Aβ-amyloid plaques after incubation with free curcumin and Ga–curcuminoid complexes (magnification 40×).

Images of the synthetic β-amyloid fibrils incubated in presence of CUR and of the Ga–curcumin complexes are gathered in Figure 3.

While the fluorescence associated to free CUR/fibrils complexes is relatively low, the fibrils incubated with the Ga-curcuminoids could be clearly visualized due to the intense fluorescence. Since an extremely low auto-fluorescence was associated with the untreated fibrils, these findings qualitatively attest the capability of the Ga–curcuminoid complexes to visualize the synthetic fibrils depositions. In particular, the fluorescence intensity appears to follow this trend: Ga(CUR)_2_^+^ ≥ Ga(DAC)_2_^+^ ≈ Ga(bDHC)_2_^+^ >> CUR.

**Figure 4:**
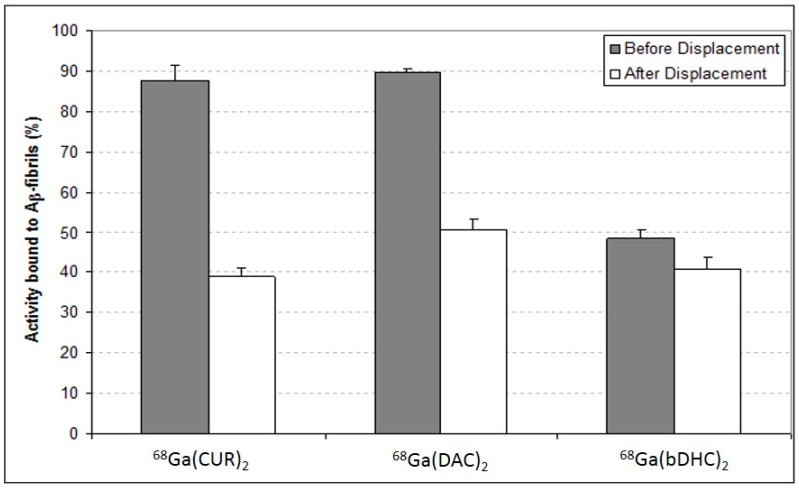
^68^Ga–curcuminoids complexes bound to the Aβ-amyloid synthetic fibrils before and after the displacement performed with a molar excess Curcumin (CUR).

To better analyse the affinity of the complexes for the Aβ-fibrils, the curcuminoids were labelled with gallium-68 and binding experiments were carried out exploiting the radioactive emission of the compounds. The percentages of the radioactivity bound to the Aβ-fibrils pellets before and after the displacement with an excess of un-labelled curcuminoids are shown in Figure 4.

Both ^68^Ga(CUR)_2_^+^ and ^68^Ga(DAC)_2_^+^ complexes are strongly retained by the fibrils (>90% bound) but quite easily displaced by the addition of an excess of correspondent curcuminoid (ca. 45% bound after displacement). Conversely, ^68^Ga(bDHC)_2_^+^ complex has a lower affinity for the fibrils (ca. 50% bound) but the compounds remain almost completely associated to the pellets also after the displacement test. 

**Figure 5:**
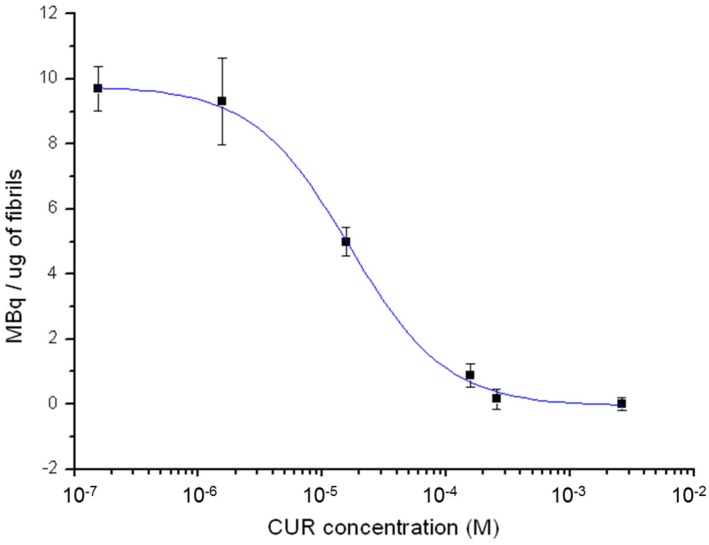
Binding affinity of ^68^Ga(CUR)_2_^+^ for synthetic Aβ-fibrils as a function of CUR concentration.

In Figure 5, the binding affinity of ^68^Ga(CUR)_2_^+^ for synthetic Aβ-fibrils as a function of CUR concentration is shown. An IC_50_ value of 1.6 × 10^−5^ M was deduced from the curve.

### 2.3. Animal Studies: Ex Vivo Visualization

**Figure 6:**
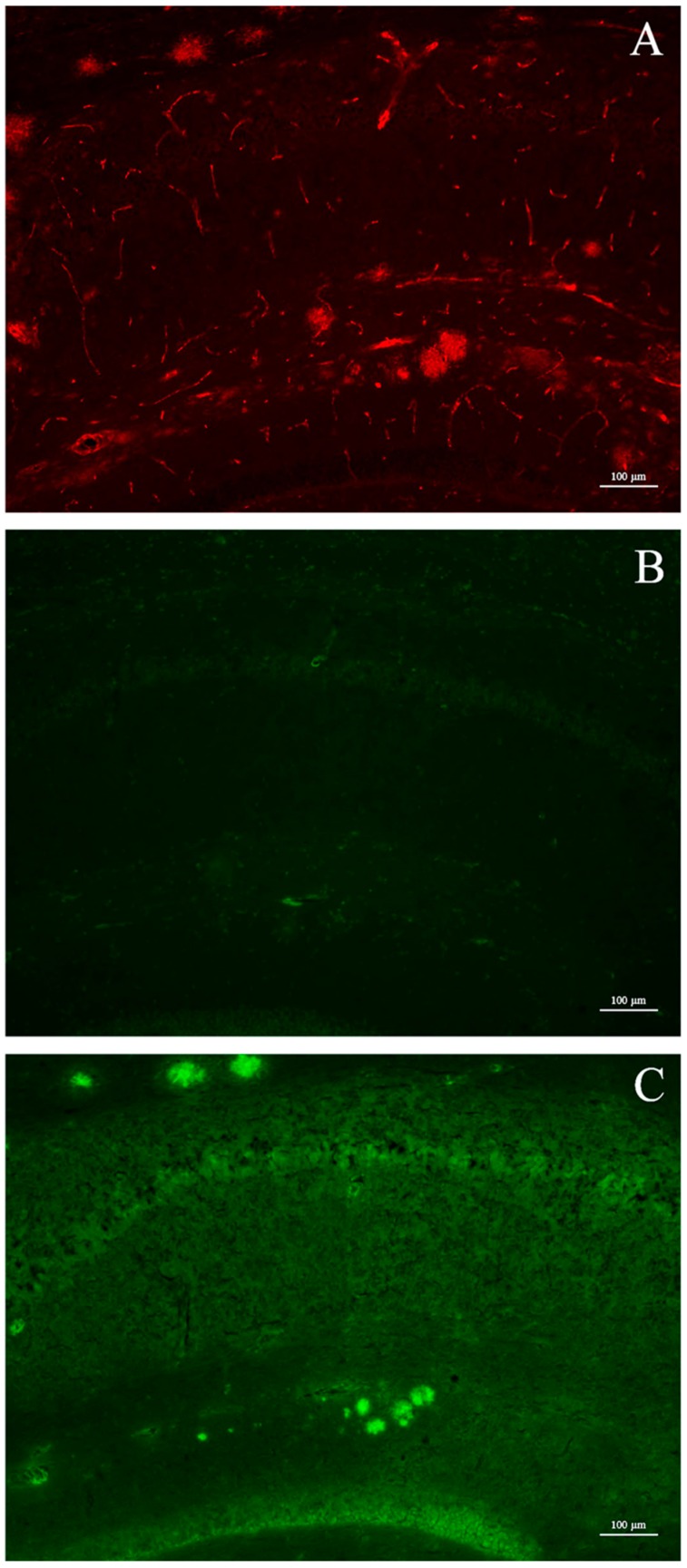
Micrographs of hippocampal sections. (**A**) Amyloid plaque deposition in hippocampus, as visualized by 6E10 immuno-staining; (**B**) Ga(CUR)_2_^+^ detection on brain section after intra-peritoneal injection. No signal is present; (**C**) Brain section after direct application of Ga(CUR)_2_^+^ solution (green: CUR fluorescence; red: rhodamine red fluorescence corresponding to amyloid β proteins).

The affinity for β-amyloid plaques in vivo of the three Ga–curcuminoid complexes was assessed using post-mortem brain cryosections from male Tg2576 mice. After injection of the compounds, the brains were explanted and the uptake was visualized exploiting their intrinsic fluorescence. Micrographs of hippocampal sections of a mice injected with Ga(CUR)_2_^+^ are shown in Figure 6. Although the formation of the amyloid plaques could be attested by the Ga(CUR)_2_^+^ direct staining (panel C) and confirmed by the 6E10 immuno-staining ex vivo (panel A), no fluorescent signal, derived from the intra-peritoneal injection, of the Ga(CUR)_2_^+^ complexes could be visualized (panel B). Comparable images were obtained for Ga(DAC)_2_^+^ and Ga(bDHC)_2_^+^ complexes (data not shown).

**Figure 7:**
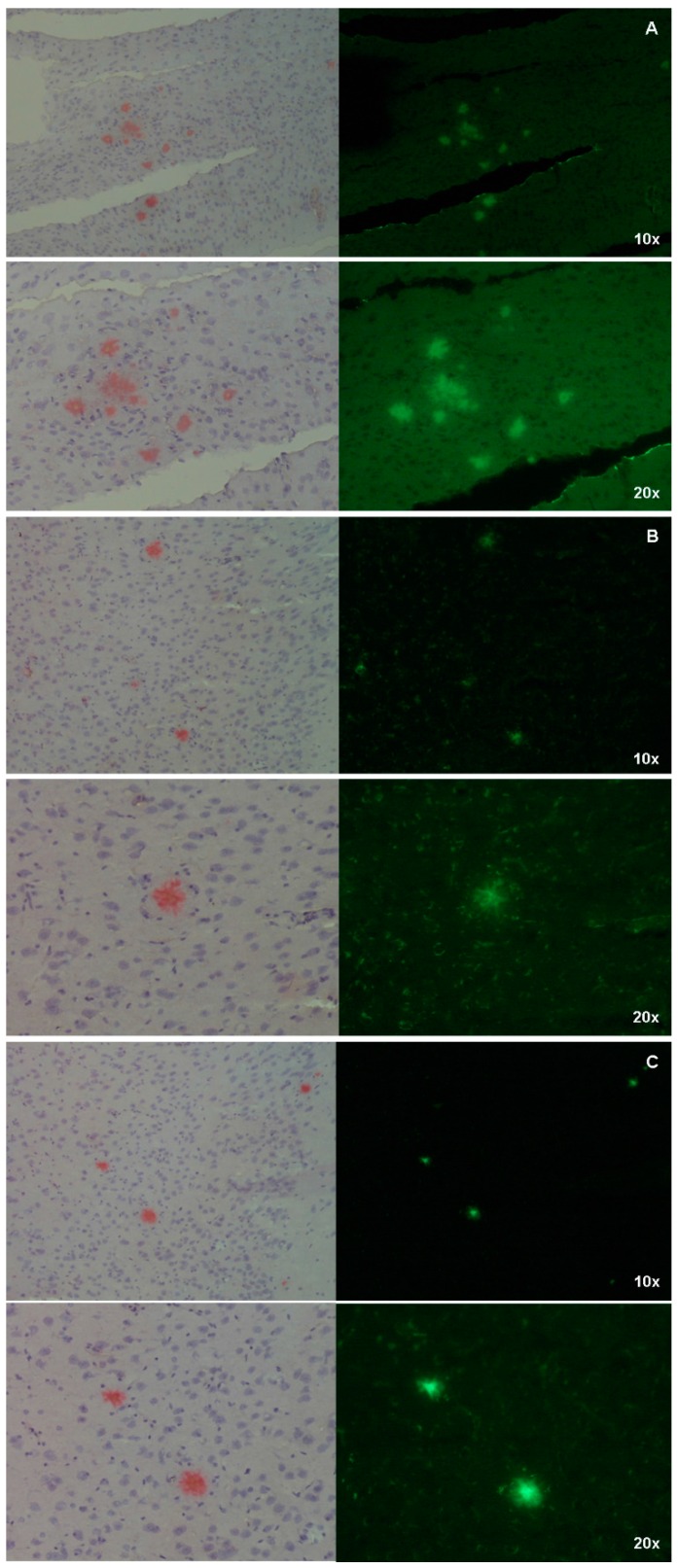
Micrographs of hippocampal sections showing Aβ-amyloid plaques stained with Ga-curcuminoids complexes (**right panels, green**) and by Congo Red as comparison (**left panels, red**). (**A**) Ga(CUR)_2_^+^; (**B**) Ga(DAC)_2_^+^; (**C**) Ga(bDHC)_2_^+^.

Conversely, the Aβ-plaques were clearly visualized by all the Ga–curcuminoid complexes when mice brain sections were directly stained with the compounds. The specificity of the uptake was attested by comparison with Congo-Red staining obtaining a high correspondence between the β-amyloids deposit visualized by the complexes and the reference compound (Figure 7, right panels).

## 3. Discussion

Alzheimer’s disease (AD) is the fifth leading cause of death in the elderly population and its incidence is a true medical emergency. AD is characterized by an extensive brain atrophy and by the presence of typical brain lesions, namely senile plaques and neurofibrillary tangles. The discovery that the accumulation of β-amyloid protein [11], resulting in plaque formation, is behind the onset of Alzheimer’s disease, allowed to identify these deposits as potential molecular targets for diagnostic and therapeutic purposes.

Diagnosis of AD is often very difficult at the early stages of the disease as it is scarcely distinguished from normal cognitive aging disorders. Moreover, while Computed Tomography and Magnetic Resonance Imaging are indispensable tools for the diagnosis of vascular dementia, the use of these techniques in degenerative dementia does not give a specific contribution at diagnosis since the differentiation between a brain atrophy and a brain cognitively unscathed is often difficult in the elderly.

On the other hand, the use of ^18^FDG-PET (fluorodeoxyglucose-positron emission tomography) was proposed as imaging techniques for AD detection since it could enable the early detection of pathophysiologic changes in patients with dementia and allow differential diagnosis [12]. However, as the ^18^FDG retention in the brain is an unspecific indicator of metabolism, it can be unbalanced, for instance, in the presence of ischemia or inflammation and could be undetectable in some people [13].

Nevertheless, due to the great potential of PET imaging, in the last years, the attention was directed to the identification and development of other highly specific radiotracers, and three ^18^F-labelled agents for imaging β-amyloid in the setting of Alzheimer’s disease were recently approved by the US Food and Drug Administration: florbetaben, flutemetamol and florbetapir [14,15,16]. However, to the knowledge of the authors, no gallium-68 labelled radiotracer has been proposed for the imaging of β-amyloid plaques so far. Herein, the uptake of three ^nat/68^Ga–curcuminoid complexes (namely, Ga(CUR)_2_^+^, Ga(DAC)_2_^+^, Ga(bDHC)_2_^+^) into amyloid fibrils and plaques has been investigated both in vitro and in vivo.

Although the curcumin structure is quite different to the Congo Red one, both the molecules share a hydrophobic bridge separating two functionalised aromatic rings. In particular, the charged sulfonate groups on the aromatic rings of Congo Red are replaced by polar methoxyl- and hydroxyl-groups on curcumin. 

In sections of tissue, Congo Red showed high affinity for amyloid and a yellow-green birefringence under crossed polarisers. Thus, Congo Red is the most popular dye used as a probe for diagnosing amyloidosis also in AD brains [17]. In the coordination with gallium, curcumin and curcuminoids act as OO bidentate ligands giving 1:2 metal to ligand complexes. Curcumin affinity for amyloid plaques was already reported by [3] suggesting that the labelling of curcumin or curcuminoids with gallium-68 or other radionuclides can trigger further advancements in the development of new biomarkers for imaging of Alzheimer’s disease. The radio-labelling was quantitative (>95%) for all the precursors yielding cationic complexes whose structure is reported in Figure 1. The in vitro experiments of affinity versus β-amyloid synthetic fibrils were performed by exploiting both the radioactivity of gallium-68 labelled compounds and the intrinsic fluorescence of the ligands. The results reported in Figure 4 show that ^68^Ga(CUR)_2_^+^, ^68^Ga(DAC)_2_^+^ and ^68^Ga(bDHC)_2_^+^ bind the fibrils with high and medium affinity, respectively. The binding of Ga(CUR)_2_^+^, Ga(DAC)_2_^+^ appears also quite specific as >50% of bound activity is displaced by an excess of free curcumin; conversely, the specificity of ^68^Ga(bDHC)_2_^+^ appears lower than the other complexes. These differences could be ascribed mainly to the lower water solubility of ^68^Ga(bDHC)_2_^+^ in respect to the other compounds that might influence the rinsing step in the affinity test. For a more quantitative result, a heterologous competitive binding assay against curcumin was performed for ^68^Ga(CUR)_2_^+^. Curcumin was chosen as an antagonist displaying high binding affinity (Ki = 3.57 nM for Aβ fibrils [6] and IC_50_ ranging from 0.68 to 361 nM depending on the studies [18]), and, hence, it is a proper competitor for this assay. In Figure 5, the resulting sigmoid fit is shown and an IC_50_ value of 1.6 × 10^−5^ M was calculated. This value is scarcely comparable with the results obtained for other radio-labelled curcumin derivatives [7,8] as well as other commercial fluorinated compounds [14,15,16], as a wide range of different inhibitors were used.

The synthetic fibrils could be clearly visualised by fluorescence microscopy after incubation with all the complexes, attesting a noticeable uptake of the compounds in the fibrils (Figure 3). Apparently, the fluorescence of all the complexes is more intense than the free curcumin one and follows this trend: Ga(CUR)_2_^+^ ≥ Ga(DAC)_2_^+^ ≈ Ga(bDHC)_2_^+^ >> CUR but this evaluation is merely qualitative and cannot be correlated with the real uptake of the compounds as many parameters might influence the fluorescent emissions. As previously reported [10], an interesting parameter to be taken into account in order to evaluate the effect of metal complexation on fluorescence emission is the quantum yield ratio (R_Φ_, defined as the ratio between the fluorescence quantum yield of the metal complex and the free ligand. CUR and DAC behave similarly with a quenching of fluorescence when gallium ion is added to give the metal complex formation (R_Φ_ ~0.5 in BSA—bovine serum albumin), while bDHC behaves the opposite with a R_Φ_ ~2 in BSA. On the other side, the fluorescence emission of gallium(III)–curcuminoid complexes decreases in the order: Ga(bDHC)_2_^+^ ≥ Ga(DAC)_2_^+^ ≈ Ga(CUR)_2_^+^. Thus, these data overtake the apparent discrepancy between the similar fluorescence emission of all gallium complexes detected by fluorescence microscopy and the in vitro experiments of affinity versus β-amyloid synthetic fibrils quantified by radioactivity emission. In fact, the lower affinity of Ga(bDHC)_2_^+^ for β-amyloid fibrils with respect to Ga(CUR)_2_^+^ and Ga(DAC)_2_^+^, which is reflected in a lower quantity binding to the fibrils in the affinity experiments, is balanced by its higher fluorescence emission.

**Figure 8:**
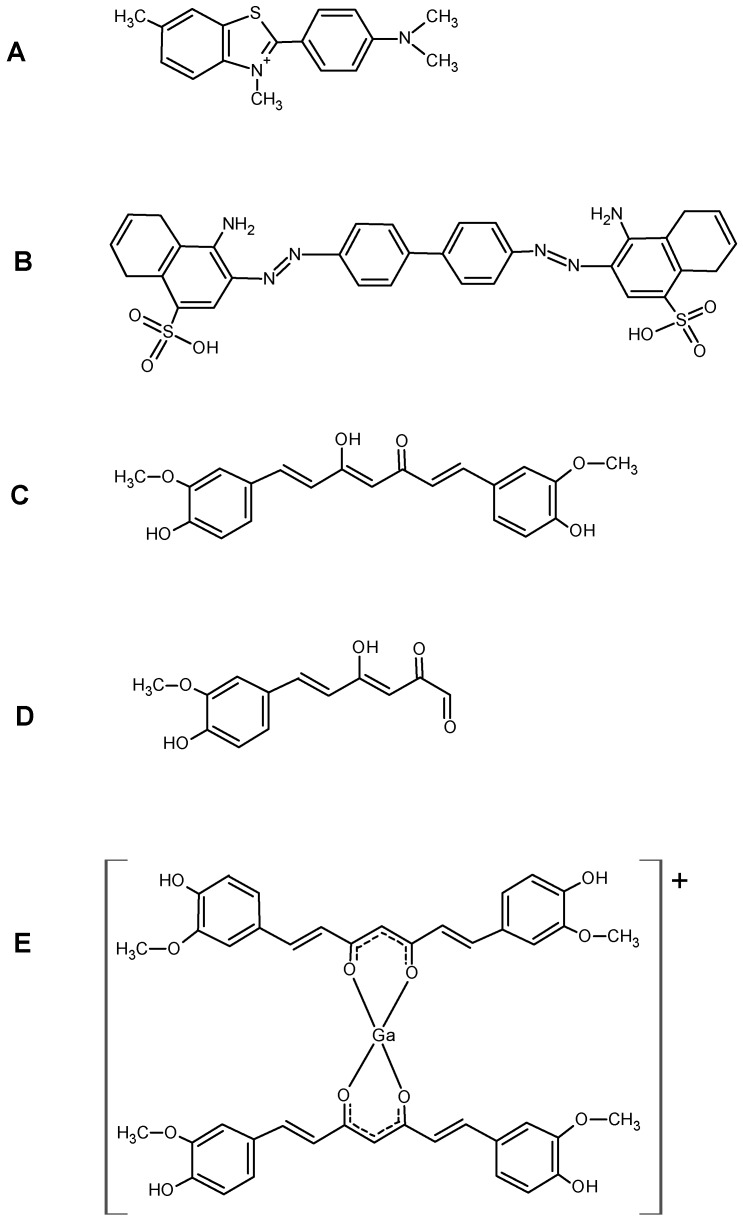
Comparison among the structures of (**A**) thioflavin; (**B**) Congo Red; (**C**) curcumin; (**D**) *trans*-6-(4′-hydroxy-3-methoxyphenyl)-2,4-dioxo-5-hexenal (curcumin metabolite); (**E**) Ga(CUR)_2_^+^ complex. The counter ion is usually chloride but, more general, depends on the media.

Similarly to free curcumin, the mechanism behind the interaction among Ga-compounds and amyloids fibrils has not been clarified yet. Recent studies show that the β-strands, whose amyloid fibrils are formed, are joined by hydrogen bridges in a rearrangement antiparallel to the direction of the fibril. The strands forms long “tapes” β-sheet, which are held together by interactions, especially electrostatic, between the side chains of amino acids [19]. It was observed that the positively charged nitrogen on the thiazole ring of thioflavin can form hydrogen bonds with amyloid fibrils [20]. Similarly, due to their unique structural features including a flexible backbone and hydrophobic nature, curcuminoid complexes might insert among the β-strand and might form hydrogen bonds with the several available H-donor and acceptor atoms. This hypothesis is reinforced by the fact that free curcumin binds more strongly to the Aβ aggregate in its more polar keto-enol form than in the di-keto form [21]. On the other hand, it cannot be excluded that, similarly to the inhibitory activity expressed for some enzymes, the affinity for the amyloids fibrils may be principally due to some curcumin degradation products rather than the intact molecule itself [22]. For a structure–activity comparison, the molecules of thioflavin, congo-red, curcumin, *trans*-6-(4’-hydroxy-3-methoxyphenyl)-2,4-dioxo-5-hexenal (curcumin metabolite) and Ga–curcuminoid complexes, are reported in Figure 8.

In addition to the studies in vitro, experiments in vivo using mice spontaneously developing Aβ-plaques (Tg2576) were conducted as well. Tg2576 mice accumulate amyloid plaques starting from 5–6 months of age and progressing until 12–14 months of age [23]. At the age of sacrifice, animals display an extensive plaque deposition in the cerebral cortex and hippocampus as shown in Figure 6, panel A. Differently from the results reported for free curcumin [3], after intra-peritoneal injection of a Ga(CUR)_2_^+^ solution, the micrographs of hippocampal sections showed no binding to plaques, resulting in the absence of fluorescent signals (Figure 6, panel B). These findings cannot be ascribed to a reduced affinity of Ga(CUR)_2_^+^ for the naturally generated amyloid aggregates with respect to the synthetic fibrils as, in a second set of experiments where the affinity of the three Ga–curcuminoid complexes was assessed by direct staining on the brain sections, the plaques were clearly visualized (Figure 6, panel C and Figure 7).

These results could be related to the impossibility of the Ga–curcuminoid complexes to cross the blood-brain barrier (BBB) and, more in general, to the low stability in physiological media of the complexes and of the curcumin like structures themselves. In fact, it was shown that free curcumin, thanks to its lipophilic nature, crosses the BBB and binds to the amyloids fibrils partially limiting their aggregation in plaques [3] but, conversely, the positive charge of the complexes may hamper the passage to the membrane and consequently limit the capability of the Ga–curcuminoid to bind the β-amyloid aggregates. 

Although DAC and bDHC (unpublished results) are more stable than curcumin itself in physiological environments [24], the complex formation slows down the degradation of curcuminoids in vitro at least in the first 2 h [25], and all the complexes showed high and comparable stability to transchelation and transmetalation when challenged with diethylenetriaminepentaacetic acid (DTPA), Fe^3+^, Cu^2+^, and Zn^2+^ solution [10]. We may not exclude the fact that fast metabolization and chemical degradation of the gallium complexes take place in vivo. Actually, it is known that curcumin is rapidly reduced to tetrahydrocurcumin and subsequently converted to ferulic acid and dihydroferulic acid in the hepatic metabolism following rapid systemic elimination [26], and this may account for the reduced bioavailability of curcumin and its derivatives after injection. It is likely to suppose that the Ga–curcuminoid complexes follow a similar metabolic path.

To enhance their bio-stability some adjuvants may be inject that are able to block the metabolic pathways responsible for curcumin metabolization. In regards to this, it is well known that piperine, an alkaloid found in black pepper, inhibits hepatic and intestinal glucuronidation, obtaining a greater bioavailability of curcumin [27]. However, with the aim of the development of a PET radiotracer, also the stability of the complexes themselves has to be taken into account since the escape of the radioactive metal core from the ligand cage would unavoidably lead to the failure of the approach. ^68^Ga(CUR)_2_^+^, ^68^Ga(DAC)_2_^+^, ^68^Ga(bDHC)_2_^+^ showed high stability in human serum but data related to their behaviour in human blood have not yet been reported [10]. Micro-PET imaging could give valuable insights to better understand both the stability issue and pharmacokinetic behaviour of this compounds in vivo in order to project a second generation of gallium-68 labelled curcuminoids.

Another reason for the lack of signal derived from the Ga–curcuminoid complexes in the Tg2576 mice brains might hail from the route of administration of the compounds. In fact, in most of the past studies, free curcumin has been administered to mouse models in the systemic circulation [3,28,29,30] or orally by gavage or supplemented chow [3,4,31,32] while, herein, the complexes were injected intraperitoneally. Although intraperitoneal administration is considered a parenteral route of delivery, substances administered intraperitoneally might be subjected to hepatic metabolism before reaching the systemic circulation because of absorption by mesenteric vessels. Moreover, absorption of substances following intraperitoneal injection is usually slower than following intravenous injection [33].

## 4. Experimental Section

### 4.1. Chemicals

Bachem (Bubendorf, Switzerland) provided us amyloid β-protein (1–40), and reagents and solvents for synthesis and characterization of the curcuminoids complexes were purchased from Sigma-Aldrich (Milan, Italy).

A 370 MBq itG ^68^Ge/^68^Ga generator was supplied by ITM (Garching, Germany) and Rotem (Leipzig, Germany) provided us metal free hydrochloric acid (0.1 M). When needed, milliQ water (resistivity 18.2 Mµ·cm) was used for preparing reagents solutions. Unless differently stated, all commercial chemicals were used without further purification. 

Curcumin[1,7-bis(4-hydroxy-3-methoxyphenyl)-1,6-heptadiene-3,5-dione](CUR), diacetylcurcumin[1,7-bis(4-acetyl-3-methoxyphenyl)-1,6-heptadiene-3,5-dione](DAC), bis-dehydroxycurcumin [1,7-bis(3-methoxyphenyl)-1,6-heptadiene-3,5-dione] (bDHC), and their related complexes with natural and gallium-68 were synthesized and characterized as previously reported [10,34].

Briefly, 5.7 mM GaCl_3_ MeOH (Methanol) solutions (2.85 µmol) were added to a 5.7 mM curcuminoids solution (5.0 µmol) in MeOH/DMSO 3:1 (bDHC) or MeOH (CUR, DAC) to prepare Gallium(III) complexes and stirring the mixture for 3 h at RT. For the synthesis of gallium-68 complexes, the curcuminoids were dissolved in acetonitrile (ACN) (DAC, bDHC) or EtOH (CUR) in order to obtain 1 mg/mL solutions and the ^68^Ge/^68^Ga generator was eluted with 4 mL of metal free 0.05 M hydrochloric acid.

Sixty nmol of curcuminoids were then added to 350 µL (about 72 MBq) of the generator eluate and subsequently were combined with 35 µL of 1.5 M sodium acetate (pH 5). The mixture was heated at 90 °C for 10 min and the completion of the labelling was assessed by UHPLC (Ultra High Performance Liquid Cromatography) or TLC (Thin-layer chromatography).

When used for in vivo experiments, the methanol solutions of Ga(CUR)_2_^+^ were evaporated to dryness and the residue was collected and dissolved in a DMSO/H_2_O solution 10/90 *v/v*. The 6E10 anti-Aβ1-16 monoclonal antibody and rhodamine Red-X-secondary antibody were purchased from Signet Laboratories (Dedham, MA, USA) and Jackson ImmunoResearch (Baltimore, PA, USA), respectively. Congo-Red staining kit was acquired from Histo-Line Laboratories (Milan, Italy).

### 4.2. Statistics

Mean values were calculated from the individual measurements and expressed at a precision of one standard deviation. Unless differently stated, all the experiments were performed in triplicate. Competitive binding assay analysis and sigmoid fitting were performed with an Origin 6.0 software (OriginLab Corporation, Northampton, MA, USA).

### 4.3. Instrumental Analysis

Tissue observation and analysis were performed with Eclipse E600 Nikon microscope equipped with an appropriate filter set (fluorescein isothiocyanate (FITC) filter set: excitation 465–495 nm, barrier filter 515–555 nm, TRITC: excitation 510–560 nm, barrier filter 590 nm). To evaluate β-amyloid plaque staining, slides were captured using a digital CCD camera Q Imaging Retiga-2000RV (Q Imaging, Surrey, BC, Canada).

A MultiScreen Separation System Harvest plate (Millipore, Milan, Italy) equipped with a vacuum pump and glass fibre plates was used for the competitive binding assay. A dose calibrator (ISOMED2000, MED Nuklear-Medizintechnik, Dresden, Germany) was employed to measure the activity. The imagesafter Congo-Red staining was observed and captured by using a Nikon Eclipse Ci optical microscope.

### 4.4. In Vitro Study

The preparation of Aβ(1–40) amyloid fibrils was performed according to methods previously published [35]. Briefly, 0.5 mg of β-amyloid protein was dissolved in 1 mL of filtered phosphate-buffered saline (PBS), pH 7.4, and magnetically stirred at 760 rpm for 3 days at RT. The mixture was centrifuged at 25,830× *g* for 15 min to separate the pellet containing Aβ fibrils from not-aggregated Aβ proteins, in suspension. The supernatant was discarded, the Aβ fibrils pellet was washed twice with PBS and subsequently was suspended in 1 mL filtered PBS. A Congo Red binding assay was performed to validate the fibrils’ formation.

To evaluate the affinity of the compounds for the synthetic amyloid fibrils, the fibrils solution was stirred in order to homogenize the suspension and aliquots of 20 µL (10 µg) were incubated for 3 h at 37 °C with (i) 400 µL of a PBS/MeOH solution (as a negative control) (ii) 400 µL of a 10 µM solution of CUR in MeOH (iii) 400 µL of a 5 µM solution of Ga–curcuminoid complexes (Ga(CUR)_2_^+^, Ga(DAC)_2_^+^, Ga(bDHC)_2_^+^) in MeOH. The samples were centrifuged at 25,830× *g* for 15 min, the supernatant was removed and pellets were washed twice with filtered PBS 1× before being suspended again in 400 µL of filtered PBS. A 100 µL of each sample was placed in a cytospin slide chamber and spun at 800 rpm for 5 min. The slides were carefully removed from the cytocentrifuge and allowed to dry prior to the visualization by fluorescence microscopy.

To evaluate the specificity of the binding between the curcuminoid complexes and the Aβ synthetic fibrils, displacement tests were performed as following. Aliquots of 70 µL (35 µg) of amyloid fibrils were incubated at 37 °C for 10 min with 500 µL (ca. 20 MBq) of ^68^Ga–curcuminoids complexes. The mixtures were centrifuged at 25,830× *g* for 15 min, the supernatants were discarded and the pellets were washed twice with filtered PBS.

Pellets were then measured in the dose calibrator. Displacement was performed by adding 500 µL of a 10 µM solution of curcumin to the ^68^Ga–curcuminoid complexes/amyloid fibrils pellets. The samples were homogenised by stirring and the procedure of incubation, centrifugation and washing described above was repeated. Finally, the samples were measured in the dose calibrator for evaluating the residual activity bound to the pellets.

Binding affinity studies to Aβ-amyloid synthetic fibrils were performed only for ^68^Ga(CUR)_2_^+^ complex by using heterologous competitive binding assay against curcumin. Aliquots of 30 µL (15 µg) of amyloid fibrils were dispensed in each well of a MultiScreen Separation System Harvest plate. Subsequently, 20 µL (1.3 MBq) of a ^68^Ga(CUR)^+^_2_ solution were added to each well followed by 100 µL of curcumin solutions at concentration ranging from 10^−3^ to 10^−7^ M in ethanol. The mixtures were incubated at 37 °C for 10 min and the bound radioactivity was collected on 1.0 µm glass fibre filters which were then rinsed three times with filtered PBS 1×. Finally, the filters were separated from the plate and measured in the dose calibrator. In order to compute the amount of ^68^Ga(CUR)_2_^+^ un-specifically bound the bottom of the wells, the procedure described was also replicated by adding the radioactive compounds to the wells in absence of fibrils. Specific uptake was calculated as MBq per µg of fibrils after subtracting the value obtained for the un-specific binding. A competitive binding curve was constructed from the data by using a sigmoid fit and the half maximal inhibitory concentration (IC_50_) was determined.

### 4.5. Animal Studies: Ex Vivo Visualization

Animal experiments were performed in 18 months old Tg2576 mice (Taconic Europe, Ejby, Denmark). The Tg2576 mice carry a trans-gene coding for the 695-amino acid isoform of the Swedish mutation of amyloid precursor protein (APP) and spontaneously develop Aβ deposits with increasing age [36]. Animal care and treatments were in agreement with the EU Directive 2010/63/EU for animal experiments and in accordance with protocols approved by the Ethical Committee of Animal Experimentation, University of Bologna. All efforts were made to minimise the number of animals used and their suffering

In the first set of experiments, Ga–curcuminoid complexes were prepared at 2 mg/mL in water solutions containing 10% DMSO and administered through a single intra-peritoneal injection of 100 μL at a final dose of 10 mg/kg. After 4 h, mice were sacrificed by anaesthetic overdose (*Iso*flurane 5%, exposure until one minute after breathing stops). The brains were quickly removed and fixed in paraformaldehyde 4% and picric acid 14% (pH 6.9) for 24 h. Subsequently, after 48 h of washing in 5% sucrose, the brains were frozen and sectioned (14 μm thick) to include the cerebral cortex and the hippocampus for the auto-fluorescence visualization. For positive control purposes, adjacent sections were stained using 6E10 anti-Aβ1–16 monoclonal antibody at a 1:1000 dilution and rhodamine Red-X-secondary antibody at a 1:100 dilution.

In the second set of experiments, mice were sacrificed without any injection of the Ga–curcuminoid and the brains were removed and treated as described above. The ex vivo affinity of the three Ga–curcuminoid complexes was assessed by direct staining on the brain sections adjacent to those used for the visualization of the amyloid plaques after intra-peritoneal injection.

The cryosections were thawed to room temperature for 15 min and then incubated for 45 min with 40 μM solutions of the compounds in DMSO [9]. The sections were washed with 40% ethanol for 1 min, following by rinsing with distilled water for 30 s and visualized by fluorescent microscopy. For positive control purposes, the same sections were stained with Congo Red by using standard laboratory procedures [37] and the correspondent regions were visualised by optical microscopy. 

## 5. Conclusions 

In this paper, the first gallium-68 labelled compounds potentially directed to the diagnosis of AD were described. Data collected showed that the curcuminoid complexes have a considerable (^68^Ga(CUR)_2_^+^, ^68^Ga(DAC)_2_^+^) or moderate (^68^Ga(bDHC)_2_^+^) affinity for both amyloid fibrils and plaques *in vitro*. Conversely, all the complexes failed in the detection of the amyloid aggregates in vivo. Hence, further modification is needed for enhancing the lipophilicity and the stability of this class of compounds before they could be used for early diagnosis of AD, however, the use of a radionuclide like gallium-68 opens up new possibilities for the use of curcumin-like structures as PET radiotracers.
